# Orthopedic infectious diseases: a survey on the composition and perceived value of an emerging subspecialty clinical service

**DOI:** 10.5194/jbji-9-161-2024

**Published:** 2024-06-12

**Authors:** Nicolás Cortés-Penfield, Don Bambino Geno Tai, Angela Hewlett

**Affiliations:** 1 Division of Infectious Diseases, University of Nebraska Medical Center, Omaha, NE, USA; 2 Division of Infectious Diseases and International Medicine, University of Minnesota, Minneapolis, MN, USA

## Abstract

We surveyed US orthopedic infectious disease (Ortho ID) specialists and surgeons ( n=54 clinicians from at least 17 institutions). Three-quarters had a dedicated clinic or inpatient service; orthopedic device-related infections were most commonly seen. All respondents highly valued Ortho ID teams for improving multidisciplinary communication, trust, access to care, and outcomes.

## 1 Introduction

Orthopedic infections are increasingly common and impose substantial mortality and morbidity. Hip and knee prosthetic joint infection (PJI) rose nearly 50 % in the United States from 2008–2018 and is predicted to cost USD 1.85 billion annually by 2030 (Patel, 2023). PJI treatment is challenging: failure rates approach ∼ 40 % when managed with debridement and implant retention and fewer than half of patients successfully complete two-stage exchange (Kurtz et al., 2022; Kunutsor et al., 2018). The 5-year mortality of PJI exceeds 20 %; among survivors, two-thirds require ambulatory aids, and a fifth can no longer live independently (Patel, 2023). Diabetes-related foot infection (DFI), which complicates ∼ 40 % of ulcers, has a similarly dire prognosis, with up to half of hospitalized DFIs leading to amputation, a 5-year mortality after major amputation exceeding 50 %, and a 1-year relapse rate of 40 % (Armstrong et al., 2020; Zhang et al., 2017; Jia et al., 2017; Richard er al., 2011). Treatment of diabetes-related infections now costs the United States almost USD 80 billion annually, similar to the cost of all cancer care (Armstrong et al., 2020).

The field of infectious diseases (ID) is diversifying into unique subspecialties as our patients grow more numerous and complex. For example, recognizing the need for an individualized, team-based approach to infection in patients undergoing organ transplantation, transplant ID emerged as a subspecialty with the founding of the American Transplantation Society (AST) in 1982 and the AST Infectious Disease Community of Practice in 2002. Today, many US fellowship programs offer dedicated pathways for transplant ID.

Management of bone and joint infections, done well, is similarly complex, requiring individualized decision-making based on a comprehensive assessment of prognostic factors to inform multidisciplinary decisions about surgical and antimicrobial management, with similarly high stakes for treatment failure (Walter et al., 2022; Nelson et al., 2023; Cortes-Penfield et al., 2023b). Orthopedic infectious diseases (Ortho ID) is now too emerging as a US ID subspecialty, with fellowship programs offering dedicated Ortho ID training pathways, a dedicated subspecialty society (the Musculoskeletal Infection Society, MSIS) shared by like-minded ID clinicians and orthopedic surgeons, and large US tertiary surgical referral centers developing dedicated teams mirroring long-standing European programs such as the UK's Bone Infection Unit at the Nuffield Orthopaedic Centre and the Interdisciplinary Unit for Orthopaedic Infections in Switzerland (Vasoo et al., 2019).

To date, there have been no studies documenting the composition and productivity of US Ortho ID groups. We performed a pilot survey characterizing US Ortho ID practice.

## 2 Methods

From 19 December 2023 to 19 January 2024, we recruited Ortho ID specialists through the mailing list of a monthly national Ortho ID case conference organized by MSIS members. We asked respondents to complete an online survey and forward the survey invitation to other Ortho ID clinicians and orthopedic surgeons. The survey for ID clinicians focused on the composition of their Ortho ID practice and the perceived value of the service, with the former presented as descriptive data and the latter assessed via a 5-point Likert scale. The survey for orthopedic surgeons focused on their perceived value of having a dedicated Ortho ID team rather than generalist ID consultants, again via Likert scale. Both groups were invited to submit open-ended feedback. The survey instrument is presented in the Supplement.

## 3 Results

There were 54 respondents, including 30 Ortho ID clinicians (a 56 % response rate) and 24 orthopedic surgeons. Respondents were highly experienced; 15 % had been in practice > 20 years, 35 % 11 to 20 years, and 24 % 6 to 10 years. Respondents came from at least 17 institutions (11 did not disclose their home institution), with a geographic distribution shown in Fig. S1 in the Supplement. One institution had three survey respondents, and three institutions had two respondents; therefore, the survey results below regarding the composition and scope of Ortho ID teams are reported using only the first ID respondent from each institution (total n=25 ), while the results regarding respondents' valuation of Ortho ID teams used the full data set.

Ortho ID teams were comprised of a median of three (IQR 2–4) ID physicians with a median of zero (IQRs each 0–1) APPs (advanced practice providers), pharmacists, and nurses. Prosthetic joint infection most often fell within Ortho ID's scope of practice, reported as “always” seen by Ortho ID by 92 % and “only if orthopedic surgery is involved” by 0 %. This was followed by fracture-related infection (84 % and 0 %, respectively), vertebral hardware infection (72 % and 16 %), native joint septic arthritis (68 % and 12 %), native vertebral osteomyelitis (56 % and 16 %), and diabetes-related foot osteomyelitis (52 % and 16 %). A minority of respondents indicated their Ortho ID teams routinely saw sacral osteomyelitis (40 %; an additional 20 % only if orthopedics was involved), necrotizing soft tissue infection (28 % and 36 %), and other surgical device infections (24 % and 16 %). Several reported other surgical services preferentially consulted their Ortho ID team, most often including plastic surgery (48 %), podiatry (44 %), neurosurgery (36 %), vascular surgery (32 %), and other services (16 %).

A total of 64 % of ID respondents had a dedicated Ortho ID clinic, with a median weekly census of 25 (IQR 13–35) patients. Of these, all indicated their clinic was scheduled at least physically and temporally adjacent to their orthopedic surgery colleague's clinic, and 60 % reported seeing patients in clinic alongside surgeons. A total of 56 % of ID respondents had a dedicated inpatient Ortho ID service with a median daily census of 10 (IQR 8–15) patients; of these, 29 % said their service also helped off-load consults from the General ID service. In total, 11 institutions (44 %) had both formal inpatient and outpatient Ortho ID teams, while 6 (24 %) had neither. Most respondents involved trainees in their Ortho ID practice, including ID fellows (72 %), orthopedic surgery residents (28 %), students (16 %), and internal medicine residents (8 %).

Both ID specialists and surgeons highly valued their dedicated Ortho ID teams. Among Ortho ID clinicians, 90 % considered improved communication with surgeons and understanding of patients' surgical care a “very important” benefit of having a dedicated Ortho ID team, 83 % said the same of building mutual trust with surgeons to increase acceptance of ID recommendations, 80 % said that facilitating timely outpatient consultations and correlated follow-up visits was a very important benefit of having a dedicated Ortho ID team, and 63 % said that focusing their clinical interests allowed them to read the literature more deeply and develop more nuanced expertise. Among the surgeons, 96 % each strongly agreed that having a dedicated Ortho ID team (versus a generalist ID consult team) improved communication and coordination with ID, improved trust and confidence in ID recommendations, and improved their patients' access to ID care; unanimously, surgeons strongly agreed that referral centers for complicated orthopedic infections should have a dedicated Ortho ID team.

Open-ended comments were offered by 14 ID clinicians and 10 surgeons; illustrative examples of these are provided in Table 1. The most common theme was surgeons' perspectives that having an Ortho ID specialist much improved their patient's care and/or should become standard. Additional comments emphasized the value of longitudinal relationships that evolve out of having a dedicated Ortho ID team and the benefits to trust and communication that follow and the value of improving coordination, continuity, and/or consistency of care. Some ID clinicians lamented that their institution had yet to develop formal dedicated Ortho ID inpatient and/or outpatient services or pointed out opportunities to synergize their work with outpatient parenteral antimicrobial therapy programs.

**Table 1:** Illustrative open responses on the value of Ortho ID teams.

From ID clinicians
“Improved communication with the surgeons and better coordination of care are our main value adds – we are consistently able to shorten [length of stay] and/or avoid admissions entirely by being able to have dedicated clinic time to follow and manage [outpatient parenteral antimicrobial therapy] and/or book urgent clinic appointments for our ortho colleagues. The surgeons have told me they really appreciate having a group they can trust to call and be confident ID management will be taken care of appropriately.”
“I think patients like seeing that I am in close communication with the surgeons. It makes them feel more confident in the plan. Also it is a more efficient use of their time to see ortho and ID on the same day.”
“Multidisciplinary, coordinated team-based care is the key to providing the highest level of patient care for patients with complicated bone and joint infections. Keeping the Ortho ID service relatively small … provides continuity and builds trust, both from our patients as well as the surgeons.”
“I feel like our Ortho ID team is invaluable given the trust and relationship that's developed between orthopedic surgeons and ID. The relationship has also helped improve ease of communication between trainees from both teams and understanding challenges in care from both a surgical and medical point of view.”
From orthopedic surgeons
“There is significant improvement in communication when drawing from a smaller pool of subspecialists in Ortho ID versus anyone in the ID division.”
“Seeing the patient with our ID colleagues improves our communication, timely care and decisions for the patients and allows us to share our concerns and process improvements in a quick and efficient manner. We are very fortunate to have the support for this at our hospital.”
“The cooperation and collaboration between ortho and ID is essential to top level care and a likely model that other centers will copy going forward.”
“The collaboration with our Ortho ID service has been one of the best quality improvement measures that have happened during my career.”

## 4 Discussion

Ortho ID is an emerging subspecialty, with over a dozen institutions across the United States identified in this survey alone as having Ortho ID specialists and nearly half of respondents working within both dedicated inpatient and outpatient Ortho ID teams. The value of multidisciplinary teams in orthopedic infection care has been repeatedly demonstrated. Retrospective studies indicate both that patients treated for osteomyelitis without involving an ID consultant are more likely to experience relapsed infection and also that patients seen by dedicated Ortho ID specialists rather than an on-call ID specialist have better outcomes (Arias Arias et al., 2015; Ziran et al., 2003). Structured rather than ad hoc multidisciplinary care (i.e., implementing a weekly case discussion) has also been associated with reductions in revision surgeries and amputations in fracture-related infection, and referral to a multidisciplinary unit for orthopedic infections versus care elsewhere has been associated with reductions in reoperation, amputation, and mortality (Rupp et al., 2023; Ferguson et al., 2021).

Some of the value of these services may be due to higher degrees of clinical expertise attained through substantially greater case volumes. The median weekly patient volume reported by this group is several-fold higher than reported in a recent survey of the larger ID community via the Emerging Infections Network, wherein 53 % and 66 % of respondents reported seeing fewer than 10 inpatient and outpatient osteoarticular infections per month, respectively (Cortes-Penfield et al., 2023a). Alternately, one might hypothesize that the Ortho ID specialist may be more up to date with the orthopedic literature, where new orthopedic infection research is predominantly published. However, rather than academic knowledge or clinical experience, our respondents pointed to longitudinal relationships between small groups of clinicians – and the trust and communication they engender – as the Ortho ID team's key value. These relationships can be built and maintained by participating in the mutual care of patients but also through collaborative research, quality improvement efforts, and shared educational initiatives and multidisciplinary case conferences.

An important limitation of this study is its recruitment from a small, self-selected group of Ortho ID specialists, likely introducing bias. Asking ID respondents to solicit survey responses from their own surgical colleagues may also have introduced bias in the results. A larger follow-up survey soliciting responses from all members of the MSIS and/or the European Bone and Joint Infectious Society (EBJIS) would be methodologically more rigorous and more fully reflect national and global Ortho ID practice. Also, since there is no generally accepted definition of an Ortho ID specialist, our Ortho ID specialists were all self-identified. A few US fellowship programs offer specialty training in Ortho ID (e.g., Mayo and Stanford); for ID clinicians who have completed fellowship, “on-the-job” training is most common. We recommend that clinicians seeking to develop Ortho ID expertise join the MSIS or EBJIS, attend their annual meetings, and participate in their communities of practice. We also encourage the MSIS and EBJIS to consider developing a core curricula and/or certification process, establishing core competencies for our subspecialty.

Ortho ID specialists can contribute diagnostic, therapeutic, and infection prevention guidance to optimize antimicrobial stewardship and outcomes, improve coordination and continuity of care, and contribute to multidisciplinary treatment with effective ID-surgeon relationships built on open dialogue and mutual respect (Vasoo et al., 2019). Tertiary referral centers with large or complex orthopedic populations should consider adding this highly valued subspecialty to their repertoire of clinical services.

## Supplement

10.5194/jbji-9-161-2024-supplement The supplement related to this article is available online at: https://doi.org/10.5194/jbji-9-161-2024-supplement.

## Data Availability

The full dataset is not publicly accessible to protect survey participants' privacy and because participants' consent for individual-level data disclosure was not obtained.

## References

[bib1.bib1] Arias Arias C, Tamayo Betancur MC, Pinzón MA, Cardona Arango D, Capataz Taffur CA, Correa Prada E (2015). Differences in the Clinical Outcome of Osteomyelitis by Treating Specialty: Orthopedics or Infectology. PLoS One.

[bib1.bib2] Armstrong DG, Swerdlow MA, Armstrong AA, Conte MS, Padula WV, Bus SA (2020). Five year mortality and direct costs of care for people with diabetic foot complications are comparable to cancer. J Foot Ankle Res.

[bib1.bib3] Cortes-Penfield N, Beekmann S, Polgreen P, Ryan K, Sekar P (2023). Current Management of Osteoarticular Infections by Infectious Diseases Physicians: Results of an Emerging Infections Network (EIN) Survey, IDWeek 2023. Open Forum Infect Dis.

[bib1.bib4] Cortes-Penfield NW, Armstrong DG, Brennan MB, Fayfman M, Ryder JH, Tan T-W, Schechter MC (2023). Evaluation and Management of Diabetes-related Foot Infections. Clin Infect Dis.

[bib1.bib5] Ferguson J, Alexander M, Bruce S, O'Connell M, Beecroft S, McNally M (2021). A retrospective cohort study comparing clinical outcomes and healthcare resource utilisation in patients undergoing surgery for osteomyelitis in England: a case for reorganising orthopaedic infection services. J Bone Joint Infect.

[bib1.bib6] Jia L, Parker CN, Parker TJ, Kinnear EM, Derhy PH, Alvarado AM, Huygens F, Lazzarini PA, Diabetic Foot Working Group (2017). Queensland Statewide Diabetes Clinical Network Incidence and risk factors for developing infection in patients presenting with uninfected diabetic foot ulcers. PLoS One.

[bib1.bib7] Kunutsor SK, Beswick AD, Whitehouse MR, Wylde V, Blom AW (2018). Debridement, antibiotics and implant retention for periprosthetic joint infections: A systematic review and meta-analysis of treatment outcomes. J Infect.

[bib1.bib8] Kurtz SM, Higgs GB, Lau E, Iorio RR, Courtney PM, Parvizi J (2022). Hospital Costs for Unsuccessful Two-Stage Revisions for Periprosthetic Joint Infection. J Arthroplasty.

[bib1.bib9] Nelson SB, Pinkney JA, Chen AF, Tande AJ (2023). Periprosthetic Joint Infection: Current Clinical Challenges. Clin Infect Dis.

[bib1.bib10] Patel R (2023). Periprosthetic Joint Infection. N Engl J Med.

[bib1.bib11] Richard JL, Lavigne JP, Got I, Hartemann A, Malgrange D, Tsirtsikolou D, Baleydier A, Senneville E (2011). Management of patients hospitalized for diabetic foot infection: results of the French OPIDIA study. Diabetes Metab.

[bib1.bib12] Rupp M, Walter N, Popp D, Hitzenbichler F, Heyd R, Geis S, Kandulski M, Thurn S, Betz T, Brochhausen C, Alt V. (2023). Multidisciplinary Treatment of Fracture-Related Infection Has a Positive Impact on Clinical Outcome – A Retrospective Case Control Study at a Tertiary Referral Center. Antibiotics (Basel).

[bib1.bib13] Vasoo S, Chan M, Sendi P, Berbari E (2019). The Value of Ortho-ID Teams in Treating Bone and Joint Infections. J Bone Joint Infect.

[bib1.bib14] Walter N, Rupp M, Baertl S, Ziarko TP, Hitzenbichler F, Geis S, Brochhausen C, Alt V (2022). Periprosthetic joint infection: patients benefit from a multidisciplinary team approach. Bone Joint Res.

[bib1.bib15] Zhang P, Lu J, Jing Y, Tang S, Zhu D, Bi Y (2017). Global epidemiology of diabetic foot ulceration: a systematic review and meta-analysis. Ann Med.

[bib1.bib16] Ziran BH, Rao N, Hall RA (2003). A dedicated team approach enhances outcomes of osteomyelitis treatment. Clin Orthop Relat Res.

