# Supplementary material for: Orthopedic infectious diseases: a survey on the composition and perceived value of an emerging subspecialty clinical service
Source: J Bone Jt Infect. 2024 Jun 12;9(3):161–5. doi: 10.5194/jbji-9-161-2024 (PMC11187702; doi:10.5194/jbji-9-161-2024)
Supplement: The supplement related to this article is available online at: https://doi.org/10.5194/jbji-9-161-2024-supplement. [file jbji-9-161-supplement.pdf]

Supplement of J. Bone Joint Infect., 9, 161–165, 2024
https://doi.org/10.5194/jbji-9-161-2024-supplement
© Author(s) 2024. CC BY 4.0 License.

JBJI Journal of Bone and Joint Infection Open Access

Supplement of

Orthopedic infectious diseases: a survey on the composition and perceived value of an emerging subspecialty clinical service

Nicolás Corté et al.

Correspondence to: Nicolás Cortés-Penfield (n.cortespenfield@unmc.edu)

The copyright of individual parts of the supplement might differ from the article licence.

Figure S1: Geographic distribution of US Ortho ID programs identified in this survey

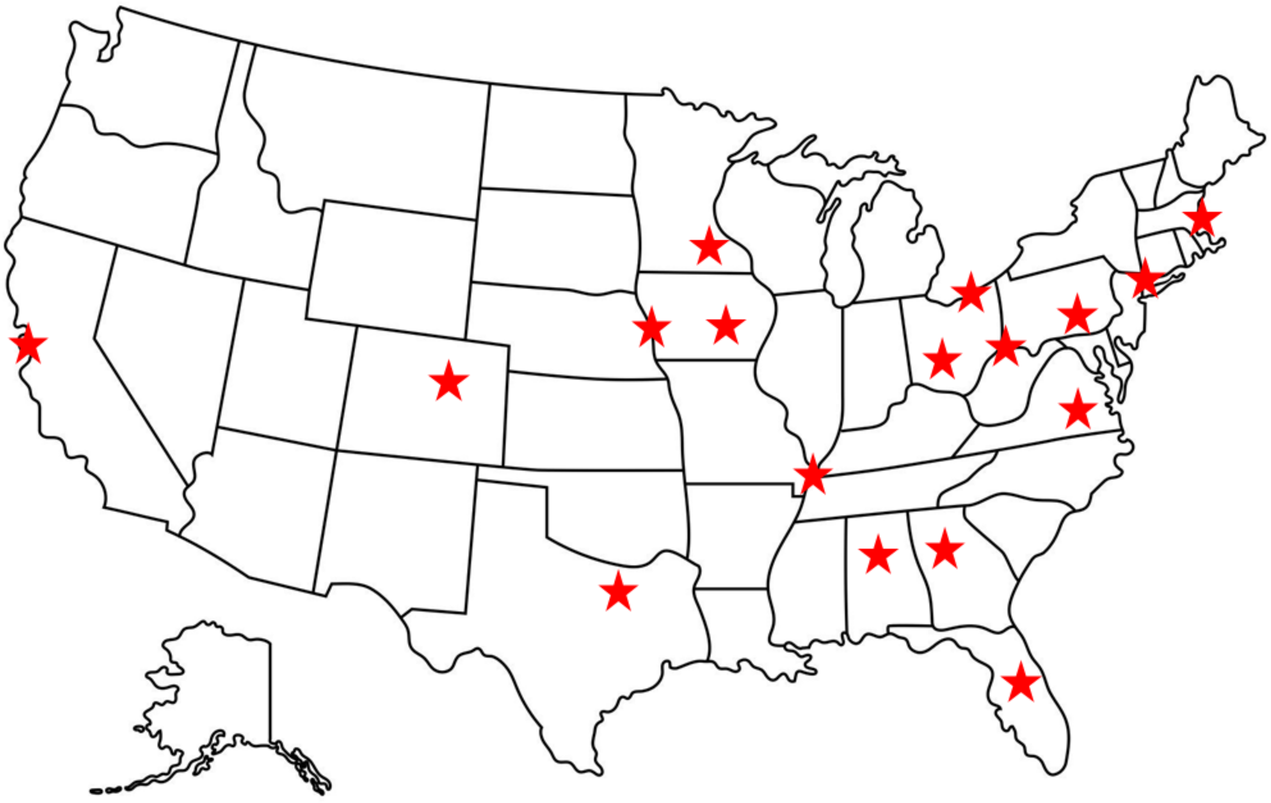

Institutions included Cleveland Clinic, Emory University, Hospital for Special Surgery, Massachusetts General Hospital, Mayo Clinic, Ohio State University, Orlando Health, Penn State Hershey Medical Center, St. Jude's Children's Research Hospital, Stanford University, University of Alabama-Birmingham, University of Colorado Medical Center, University of Iowa Health Care, University of Nebraska Medical Center, UT-Southwestern Medical Center, Virginia Commonwealth University, and West Virginia University of Medicine. **Note:** Not all respondents chose to disclose their home institutions.

Survey for Ortho ID specialists:

1. What is the composition of your Ortho ID team? Include all **infectious disease** clinicians who have a substantial inpatient or outpatient commitment dedicated to caring specifically for patients with bone and joint infections.

# of Attending Physicians [numeric dropdown list ranging 0-10]

# of Advanced practice practitioners [numeric dropdown list ranging 0-10]

# of Pharmacists [numeric dropdown list ranging 0-10]

# of Nurses [numeric dropdown list ranging 0-10]

Any other team members (if yes, please describe): [FREE TEXT RESPONSE]

2. Which patient problems fall under the purview of your Ortho ID team? (i.e. consults that usually go to the Ortho ID service rather than other ID inpatient teams or clinics)

- Prosthetic joint infection [yes/only if ortho is involved/no]
- Fracture-related infection [yes/only if ortho is involved/no]
- Vertebral hardware infections [yes/only if ortho is involved/no]
- Other surgical device-related infections (e.g. deep brain stimulator infections) [yes/only if ortho is involved/no]
- Diabetes-related foot osteomyelitis [yes/no/only if ortho is involved]
- Native vertebral osteomyelitis [yes/no/only if ortho is involved]
- Sacral osteomyelitis [yes/no/only if ortho is involved]

3. Do you have a dedicated Ortho ID inpatient service? [yes/no]

2a. What is the average census of your inpatient Ortho ID service? [Numeric dropdown list ranging 0-20]

2b. How many inpatient facilities does your Ortho ID team cover? [Numeric dropdown list ranging 0-10]

2b. Does your Ortho ID inpatient service serve any other functions? (e.g. General ID team overflow); if yes, please describe: [FREE TEXT RESPONSE]

4. Do you have a dedicated Ortho ID outpatient clinic? [yes/no]

4a. What would you estimate to be the average weekly outpatient volume (# of unique patients) of the Ortho ID clinic? [numeric response]

4b. Is your clinic co-located with one or more orthopedic surgeons? (i.e. patients are seen alongside the surgeon) [yes/no]

4c. Is your clinic coordinated with one or more orthopedic surgeons? (i.e. scheduled immediately before or after the surgeon's clinic at the same or a nearby location) [yes/no]

5. Do trainees rotate through your Ortho ID clinic or inpatient service? [check all that apply]

- Our Ortho ID team does not work with trainees
- Infectious disease fellows
- Internal medicine residents
- Orthopedic surgery residents
- Students (e.g. medical, pharmacy, NP/PA, etc)
- Other [please describe]

6. Do you have a regular Ortho ID conference to discuss challenging cases or new research with the surgeons and/or ID colleagues? [Yes, attended by both ID clinicians and surgeons/Yes, but not attended by our surgeons/no]

7. Do other surgical services preferentially utilize your Ortho ID team versus other ID teams?

Vascular surgery [yes/no]

Plastic and reconstructive surgery [yes/no]

Neurosurgery [yes/no]

Podiatry [yes/no]

Other (please describe): [FREE TEXT RESPONSE]

8. Which of the following do you consider key benefits/value of having a dedicated Ortho ID team?

Building mutual trust with surgeons makes them more likely to accept my recommendations [5pt Likert scale: “Very important”/”Important”/”Moderately important”/”Slightly important”/“Not important”]

Improved communication with surgeons helps me understand the surgical aspects of my patients’ care [5pt Likert scale: “Very important”/”Important”/”Moderately important”/”Slightly important”/“Not important”]

Focusing my clinical interests has helped me read the literature more deeply and develop a more nuanced approach to complicated orthopedic infections [5pt Likert scale: “Very important”/”Important”/”Moderately important”/”Slightly important”/“Not important”]

My patients have better access to ID care (i.e. timely outpatient consultations and correlated follow-up visits) [5pt Likert scale: “Very important”/”Important”/”Moderately important”/”Slightly important”/“Not important”]

9. Does your Ortho ID service receive any direct or indirect assistance (e.g. salary support, administrative support, or retainer) from the Department of Orthopaedic Surgery? [yes/no]

9a. If yes, please describe: [FREE TEXT RESPONSE]

10. Do you have any further comments about building a dedicated orthopedic ID team or the value of having such a team? [FREE TEXT RESPONSE]

Survey for orthopedic surgeons who work with Ortho ID specialists:

1. **Having a dedicated Ortho ID team (versus a generalist ID consult service) improves my communication & coordination with ID** [5pt Likert scale: “Strongly disagree”/”disagree”/”Neither agree nor disagree”/”Agree”/”Strongly agree”]

2. **Having a dedicated Ortho ID team (versus a generalist ID consult service) improves my trust and confidence in recommendations from ID** [5pt Likert scale: “Strongly disagree”/”disagree”/”Neither agree nor disagree”/”Agree”/”Strongly agree”]

3. **Having a dedicated Ortho ID team (versus a generalist ID consult service) improves my patients’ access to timely ID follow-up** [5pt Likert scale: “Strongly disagree”/”disagree”/”Neither agree nor disagree”/”Agree”/”Strongly agree”]

4. **Referral centers for complicated orthopedic infections should have a dedicated Ortho ID team (versus a generalist ID consult service).** [5pt Likert scale: “Strongly disagree”/”disagree”/”Neither agree nor disagree”/”Agree”/”Strongly agree”]

5. **Please share any additional comments you have about the value of a dedicated ortho ID team or about collaborating with ID specialists who do vs do not specialize in bone and joint infections:** [FREE TEXT RESPONSE]
